# A rat-based preclinical platform facilitating transcatheter hepatic arterial infusion in immunodeficient rats with liver xenografts of patient-derived pancreatic ductal adenocarcinoma

**DOI:** 10.1038/s41598-024-61142-y

**Published:** 2024-05-08

**Authors:** Masanori Ozaki, Ken Kageyama, Kenjiro Kimura, Shinpei Eguchi, Akira Yamamoto, Ryota Tanaka, Takehito Nota, Hiroki Yonezawa, Hideyuki Nishiofuku, Yuki Sakai, Naoki Tani, Atsushi Jogo, Mizue Terai, Takami Sato, Takeaki Ishizawa, Yukio Miki

**Affiliations:** 1 Department of Diagnostic and Interventional Radiology, Graduate School of Medicine, Osaka Metropolitan University, 1-4-3, Asahimachi, Abenoku, Osaka, 5458585 Japan; 2 Department of Hepato-Biliary-Pancreatic Surgery, Graduate School of Medicine, Osaka Metropolitan University, 1-4-3, Asahimachi, Abenoku, Osaka, 5458585 Japan; 3 Department of Diagnostic and Interventional Radiology, Nara Medical University, 840 Shijocho, Kashihara, Nara 6348521 Japan; 4 Department of Medical Oncology, Sidney Kimmel Cancer Center, Thomas Jefferson University, 1015 Walnut Street, 1024 Curtis Building, Philadelphia, PA 19107 USA

**Keywords:** Patient-derived tumor xenograft, Pancreatic ductal adenocarcinoma, Liver metastasis, Hepatic arterial infusion, Cisplatin, Immunodeficient rat, Cancer models, Metastasis, Cancer, Pancreatic cancer

## Abstract

Liver metastases from pancreatic ductal adenocarcinoma (PDAC) are highly fatal. A rat-based patient-derived tumor xenograft (PDX) model is available for transcatheter therapy. This study aimed to create an immunodeficient rat model with liver xenografts of patient-derived primary PDAC and evaluate efficacy of hepatic arterial infusion chemotherapy with cisplatin in this model. Three patient-derived PDACs were transplanted into the livers of 21 rats each (totally, 63 rats), randomly assigned into hepatic arterial infusion, systemic venous infusion, and control groups (n = 7 each) four weeks post-implantation. Computed tomography evaluated tumor volumes before and four weeks after treatment. Post-euthanasia, resected tumor specimens underwent histopathological examination. A liver-implanted PDAC PDX rat model was established in all 63 rats, with first CT identifying all tumors. Four weeks post-treatment, arterial infusion groups exhibited significantly smaller tumor volumes than controls for all three tumors on second CT. Xenograft tumors histologically maintained adenocarcinoma features compared to original patient tumors. Ki67 expression was significantly lower in arterial infusion groups than in the other two for the three tumors, indicating reduced tumor growth in PDX rats. A liver-implanted PDAC PDX rat model was established as a rat-based preclinical platform. Arterial cisplatin infusion chemotherapy represents a potential therapy for PDAC liver metastasis.

## Introduction

Pancreatic ductal adenocarcinoma (PDAC) is the fourth leading cause of cancer-related death, accounting for 50,550 deaths per year in the United States^1^. This malignancy is highly devastating, with an overall 5-year survival rate of approximately 5%^2^. Although surgical resection offers a potential cure, only a small number of patients are eligible for surgery at the time of diagnosis^1^. PDAC metastasis poses a significant challenge^3^. The liver is the most common site of PDAC metastasis, with approximately half of cases involving liver metastases with an associated short life expectancy^4^. One clinical approach to treat liver metastases from PDAC is chemotherapy. However, the prognosis remains limited even with major chemotherapy regimens for pancreatic cancer, such as FOLFIRINOX (folinic acid, fluorouracil, irinotecan, and oxaliplatin) or gemcitabine plus nab-paclitaxel^5^. There is a critical need for methods to identify optimal chemotherapy regimens for PDAC liver metastases.

Transcatheter hepatic arterial infusion chemotherapy has been used for liver malignancies such as hepatocellular carcinoma and colorectal liver metastases. The rationale for hepatic arterial infusion chemotherapy is that arterial infusion of drugs, rather than venous infusion, increases the liver tumor uptake of the drug^6^. The delivery of chemotherapeutic agents directly into the hepatic artery provides a higher concentration of drugs to the tumor site, potentially enhancing tumor responses. In addition, transcatheter hepatic arterial infusion may reduce toxicity associated with systemic chemotherapy. Previous studies have reported the efficacy of pancreatic arterial infusion using cisplatin in patients with inoperable advanced primary PDAC^7,8^. However, no consensus has been reached on appropriate regimens of hepatic arterial infusion chemotherapy for PDAC liver metastases.

In recent decades, animal-derived tumor-bearing models and human tumor cell lines have been utilized in the initial stages of basic cancer research to develop new therapeutic strategies. However, such models do not accurately reflect the clinical pathological environment of human tumors and cannot be directly applied in clinical practice, because the original tumor characteristics are changed owing to numerous repeated passages^9–12^. Several severely immunodeficient animals have been developed to improve the efficiency of human tumor cell transplantation and have facilitated the creation of patient-derived tumor xenograft (PDX) animal models^13,14^. PDX animal models carry tumor cells from patients, retaining the principal histological and genetic characteristics of patient-derived tumors^15^. PDX models promote drug development and aid in selecting personalized treatments based on individual molecular characteristics, thereby advancing precision medicine^16^.

Overall, we hypothesized that hepatic arterial infusion chemotherapy could be employed in liver-implanted PDX models to mimic real patient treatment. A key advantage of this model is the provision of an opportunity to create personalized therapy that finds the best anticancer drug for the patient in the PDX model, in advance of the development of PDAC liver metastases. To simulate these treatments in a PDX model, the use of large animals and invasive treatment methods is crucial. Previous studies have established a liver-implanted PDX rat model using primary PDAC, and developed transcatheter-based therapy for the rat liver^17,18^. The liver-implanted PDX rat model is well-suited for evaluating transcatheter therapy via the hepatic artery. Although the use of only the common anticancer drug, cisplatin, has been insufficient to evaluate treatment efficacy in PDAC, previous clinical trials have shown promising results^19,20^.

The purpose of this study was to develop a severely immunodeficient rat model with patient-derived primary PDAC xenografted into the liver, and to assess the treatment efficacy of transcatheter hepatic arterial and transvenous infusion with cisplatin in this model.

## Methods

### Ethics

Written informed consent was obtained from all patients for this research according to the protocol approved by the Institutional Review Board (approval no. 4376). These experiments were performed according to the “Guidelines of the Public Health Service Policy on the Humane Use and Care of Laboratory Animals.” The animal experimental protocol was approved by the Institutional Animal Care and Use Committee of our facility (approval nos. 18065 and 22053). All animal experiments were reported in accordance with the ARRIVE guidelines.

### Patient-derived PDAC samples

Human tumor samples (X0) were obtained from patients with primary PDAC who had undergone surgery at our facility between August 2019 and April 2020. Tumor samples from three patient-derived PDACs (PK1, PK9, and PK14; the letters “PK” were used to assign serial numbers to primary pancreatic cancer tissues removed from patients at our facility) were used in this study.

### Animals

Interleukin 2 receptor γ chain (IL2Rg) knockout X-linked severe combined immunodeficiency (X-SCID) rats (University of Tokyo) were used in this study^21^. Rats were kept in a sterile environment. During the in-life period, rats were maintained on a 12-h light/dark cycle and were supplied with ad libitum access to autoclaved food (Oriental CE-2 pellet diet, Oriental Yeast, Tokyo, Japan) and tap water. General condition was observed by measuring body weight and food consumption once a week throughout the in-life period. Anesthesia in rats was initially induced with 3% isoflurane, and then maintained with 1–2% isoflurane for implantation, computed tomography (CT), and interventional therapies. Rats were euthanized by CO_2_ inhalation following anesthesia with 5% isoflurane for induction.

### Cryopreservation, thawing procedure and passage

Resected specimens were washed twice with sterile phosphate-buffered saline (PBS) and cut into 5-mm cubes for implantation. These 5-mm cube samples were transferred into sterile cryotubes containing Cellbanker 1plus (Zenyaku, Fukushima, Japan) and subsequently placed in a − 80 °C freezer until use. For thawing, the cryotubes were placed in a 37 °C water bath until no longer frozen. The tumor samples were then kept on ice until implantation. After thawing, tumor samples were implanted into the liver of the recipient rats, and then transplanted into the liver of other recipient rats for a few passages until the end of the experiments. After successful engraftment in first-generation rats (X1), the xenograft tumors were removed 12 weeks later. Specimens resected from X1 rats were washed twice with sterile PBS and cut into 5-mm cubes. Some pieces were cryopreserved in a − 80 °C freezer using Cellbanker 1plus. Some pieces were transplanted into the livers of second-generation rats (X2) within 2 h of resection from X1 rats, without cryopreservation. X2 and later rats were sacrificed at 12 weeks after implantation, similar to X1 rats. To maintain PDX tumors in rats, tumors obtained from X1 rats were serially transplanted into the next cohort of rats (X2 and later). In this study, generation X3 to X9 rats were used for the experiment. To minimize structural changes between successive generations, PDX tumors within three consecutive generations were used for each of the three patient-derived PDACs in this study (X7–9 tumors in PK1; X4–6 tumors in PK9; and X3–5 tumors in PK14). The resected tumors were evaluated using hematoxylin and eosin (HE) staining and immunohistochemistry.

### Transplantation

PDAC tumors were transplanted into the livers of 6–8-week-old rats. The rats were kept under sterile conditions during transplantation. After placing the rats in the supine position under anesthesia, the abdomen was extensively disinfected with 70% ethyl alcohol, and a 2-cm lateral incision was made in the epigastric region. Concerning liver transplantation, the left lobe of the liver was maneuvered outside the body using a cotton swab, and then placed and fixed on gauze. The surface of the liver was incised horizontally using a No. 11 scalpel blade (AD Surgical, Sunnyvale, CA, USA) to form a pocket in the liver parenchyma without cutting any major vessels^22–24^. A 2-mm cube tumor sample was implanted into the liver pocket, and then the incision site was sealed using absorbable hemostatic material (SURGICEL, Johnson and Johnson, New Brunswick, NJ, USA) to stop the bleeding. The left lobe of the liver was returned to the abdominal cavity. The incision was closed in layers with 3–0 absorbable sutures (PDSPLUS, Johnson and Johnson).

### CT imaging

Liver tissues and liver tumors in rats were visualized on contrast-enhanced micro-CT using contrast agent (Viscover ExiTron nano 12000, Miltenyi Biotec, Bergisch-Gladbach, Germany)^25^. This contrast agent is an alkaline earth-based nanoparticulate contrast agent specifically formulated for preclinical CT^26^. The contrast agent was administered to rats via the tail vein as a bolus injection of 200 μL of ExiTron nano 12000. The rats were imaged 4 h after the injection. CT was performed using a micro-CT scanner (Latheta LCT-200, Hitachi Aloka Medical, Tokyo, Japan). The X-ray tube was operated using the following settings: tube voltage, 50 kV; tube current, 0.5 mA; slice interval, 240 μm; and CT scan time, 7–12 min. A tumor was defined as a non-enhancing lesion > 1 mm in diameter in the liver. Using approximately 150–200 CT images of samples, the shortest and longest diameters of the nodules were measured by two interventional radiologists using an image viewer (OsiriX version 12.0, Pixmeo, Bernex, Switzerland). Tumor volume was calculated using the following formula: volume = W^2^ × L/2, with W being the shortest diameter and L being the longest diameter^27^.

### Interventional procedure

**Figure 1:**
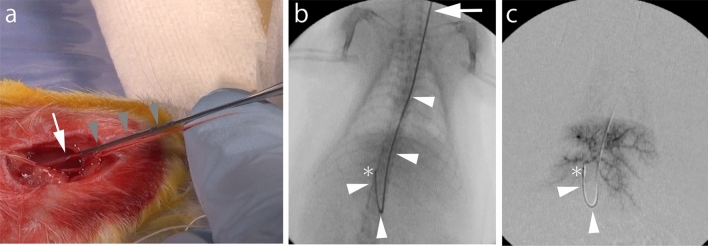
Interventional procedure. (**a**) 22-gauge intraarterial catheter (gray arrowheads) is punctured into the left common cervical artery (white arrow). (**b**) A 1.6-Fr micro-catheter with a 0.014-inch guidewire (white arrowheads) are inserted into the proper hepatic artery (asterisk). The left common cervical artery is located at white arrow. (**c**) Digital subtraction angiography via the proper hepatic artery (asterisk). Intrahepatic arterial blood vessels were depicted. A 1.6-Fr micro-catheter is indicated by white arrowheads.

After placing the rats in the supine position under anesthesia, the left neck was extensively disinfected with 5% povidone-iodine solution, and a 2-cm longitudinal incision was made in the left cervical region. Subsequently, the skin on both sides of the incision was separated, and a blunt-dissection was made to expose the left common cervical artery^18^. The left common cervical artery was cannulated using a 22-gauge intraarterial catheter (Supercath Z 20G, Medikit, Tokyo, Japan) (Fig. 1a). A 1.6-Fr micro-catheter (Tokai Medical, Aichi, Japan) and a 0.014-inch guidewire (Meister S14, Asahi Intecc, Aichi, Japan) were inserted into the arterial lumen (Fig. 1b). The microcatheter was subsequently advanced into a proper hepatic artery. Intraarterial catheterizations were performed under X-ray fluoroscopic guidance by interventional radiologists. Iodixanol contrast medium (Omnipaque; GE Healthcare Japan, Tokyo, Japan) was delivered through the catheter to visualize the hepatic vasculature. Digital subtraction angiography was performed to confirm the appearance of blood vessels (Fig. 1c). Transcatheter hepatic arterial infusion chemotherapy using a solution of 0.5 mg cisplatin (IA-call, Nihonkayaku, Tokyo, Japan) (1 mg/kg body weight) dissolved in 0.5 ml saline was performed via the 1.6-Fr micro-catheter for 3 min. After arterial infusion chemotherapy, the intraarterial catheter was removed, the left common cervical artery was ligated, and the neck incision was closed using uninterrupted sutures.

For systemic venous infusion, transvenous infusion chemotherapy using the same dose of cisplatin was performed via the tail vein for 3 min.

### Experimental workflow

**Figure 2:**
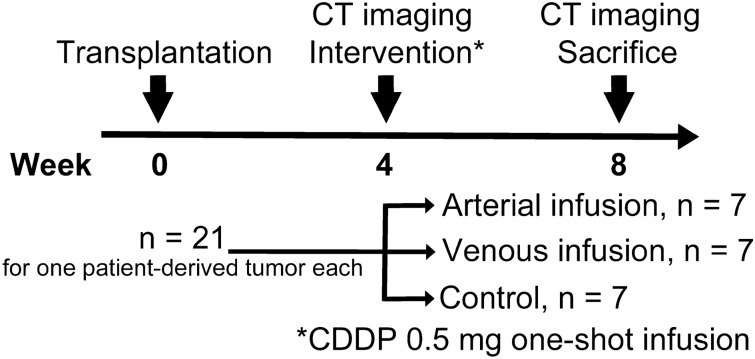
Experimental workflow.

A schematic of the experimental workflow is shown in Fig. 2. Three patient-derived tumors were transplanted into the livers of 21 rats each. In total, 63 rats were used in this study. Four weeks after transplantation, CT was performed on tumor-bearing rats to confirm the establishment of transplanted patient-derived PDAC in the liver. The 21 rats were randomly assigned into three treatment groups (n = 7 each): hepatic arterial infusion, systemic venous infusion, and control. Three types of treatment were performed for rats assigned to these groups. After treatment, rats were observed for a 4-week follow-up period. At four weeks after intervention, CT was performed to again measure tumor volume. Consequently, tumor volumes were evaluated twice: before treatment and at 4-weeks after treatment. Rats were humanely sacrificed after completion of the entire experiment and tumors were removed for pathological analysis.

### Pathology and immunohistochemistry

Following the euthanasia of the rats, livers were excised and fixed in 10% phosphate-buffered formalin. Patient-derived PDACs were sliced in the middle and slices were dehydrated and embedded in paraffin. Patient-derived PDACs were stained with HE. Using HE-stained slides, two experimenters evaluated the histopathological features of adenocarcinoma with comparisons between original tumors and xenografted tumors. Immunohistochemical analysis of Ki67 was performed using rabbit Ki67 (1:400; Cell Signaling Technology, Danvers, MA, USA) on paraffin-embedded sections to measure cell proliferative capacity^28^. Five fields of tumor tissues from a single specimen were arbitrarily photographed at 400 × magnification. Ki67-positive cells were highlighted and extracted from the five photos, and the percentage of Ki67-positive cells in each image was calculated using ImageJ software (National Institutes of Health, Bethesda, Maryland, USA)^29,30^. To increase the reliability of the calculated data, the maximum and minimum values were removed and the three intermediate values were used for analysis. The mean percentage of tumor cells staining positive for Ki67 in the three fields was calculated.

### Statistics

All values are reported as the mean ± standard deviation. Continuous data, such as tumor volume and immunostaining area, were analyzed using the nonparametric unpaired Mann–Whitney U-test. For these analyses, values of *p*-value < 0.05 were considered statistically significant. All statistical analyses were performed using Prism version 9 (GraphPad, La Jolla, CA, USA).

## Results

### Establishment of the rat liver PDX model

Patient-derived tumor specimens were successfully transplanted into the livers of 63 rats without failure. No perioperative deaths of rats were observed during this study. No complications from transplantation occurred. Findings from contrast-enhanced CT performed for all 63 rats at 4 weeks after tumor implantation showed that all xenografted tumors were engrafted as solitary nodules in the liver (engraftment rate, 100%). Abdominal dissemination was not observed in any rats. All rats were able to be observed up to 8 weeks post-tumor implantation. All rats were sacrificed at 8 weeks post-tumor implantation, just after the second CT. All rats continued to gain weight from the beginning to the end of the 8-week experimental period, showing no decrease in food intake.

### Interventional procedure for the PDX model

Transcatheter arterial therapy and venous infusion therapy successfully completed in all assigned rats without failure. No complications were identified in the arterial or venous infusion groups for 4 weeks after the intervention. All rats survived for 4 weeks after the intervention.

### Tumor evaluation

**Figure 3:**
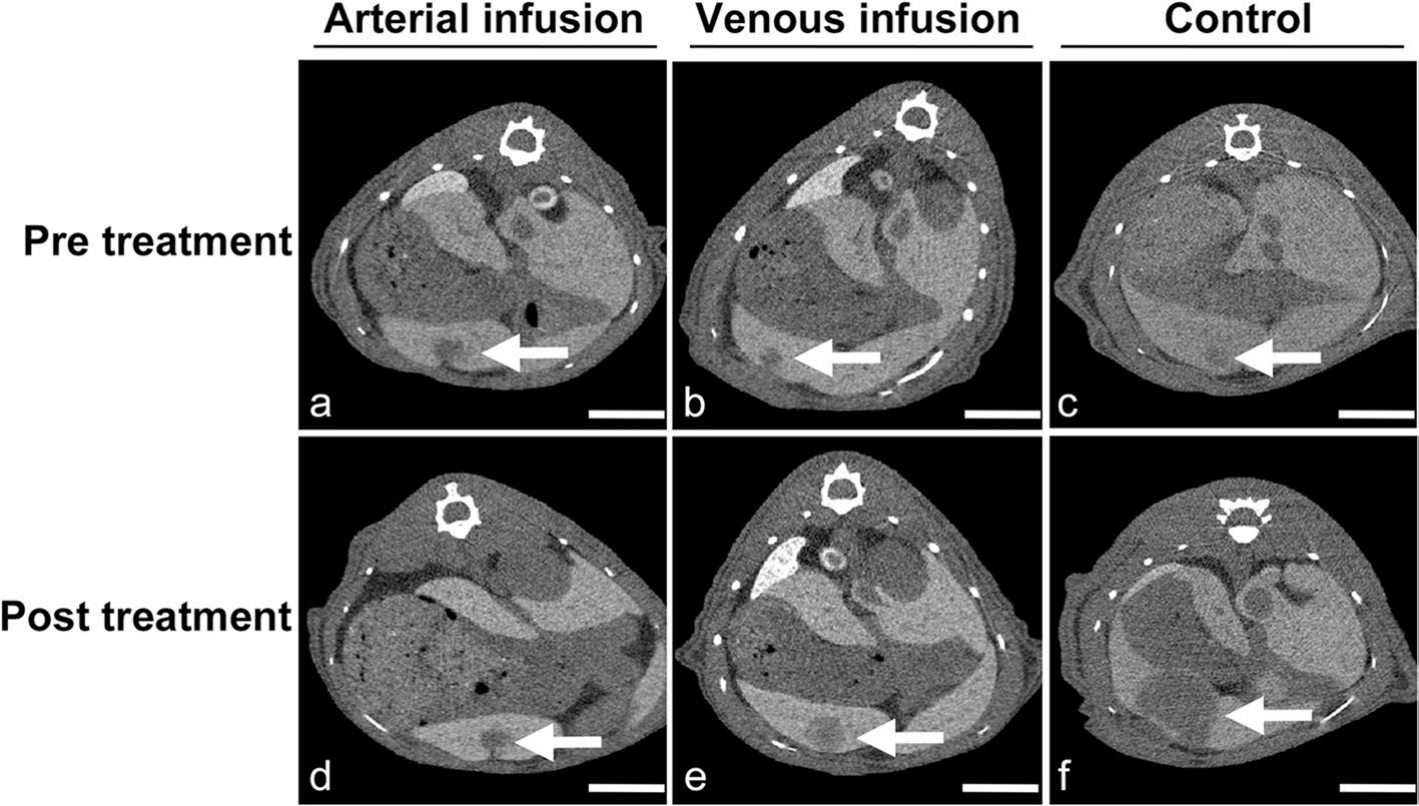
Three representative examples of CT images of arterial infusion, venous infusion, and control groups at pre-treatment and at 4-weeks after treatment using patient-derived xenograft tumors from PK14. *Scale bar* 10 mm. (**a, d**) Arterial infusion group. (**b, e**) Venous infusion group. (**c, f**) Control group.

**Figure 4:**
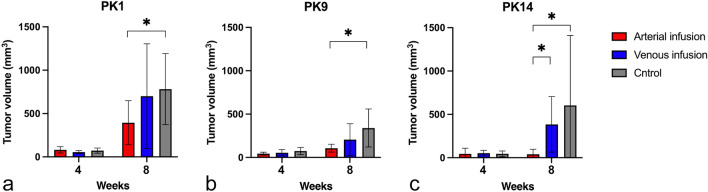
Mean tumor volume at pre-treatment and at 4 weeks after treatment for three types of patient-derived tumors. (**a**) PK1. (**b**) PK9. (**c**) PK14. *p-value < 0.05.

Patient-derived tumors from the three cases were implanted in 21 rats each, and then the rats were assigned into three groups (n = 7 each). Before treatment, no significant differences in tumor volume were observed on CT among the three groups (Figs. 3 and 4). Tumor volume was significantly smaller in the arterial infusion groups than in the control groups for all three tumors (PK1, *p* = 0.038; PK9, *p* = 0.038; PK14, *p* = 0.026) (Figs. 3 and 4), but showed no significant difference between the venous infusion and the control groups. Significant inhibition of tumor growth in terms of tumor volume was observed between the arterial and control groups for all three tumors. In particular, the tumor volume in the PK14 arterial infusion group was reduced post-treatment compared to pre-treatment. With PK14, the tumor volume was also significantly smaller in the arterial infusion group than in the venous infusion group (*p* = 0.007) (Fig. 4c).

### Pathology

**Figure 5:**
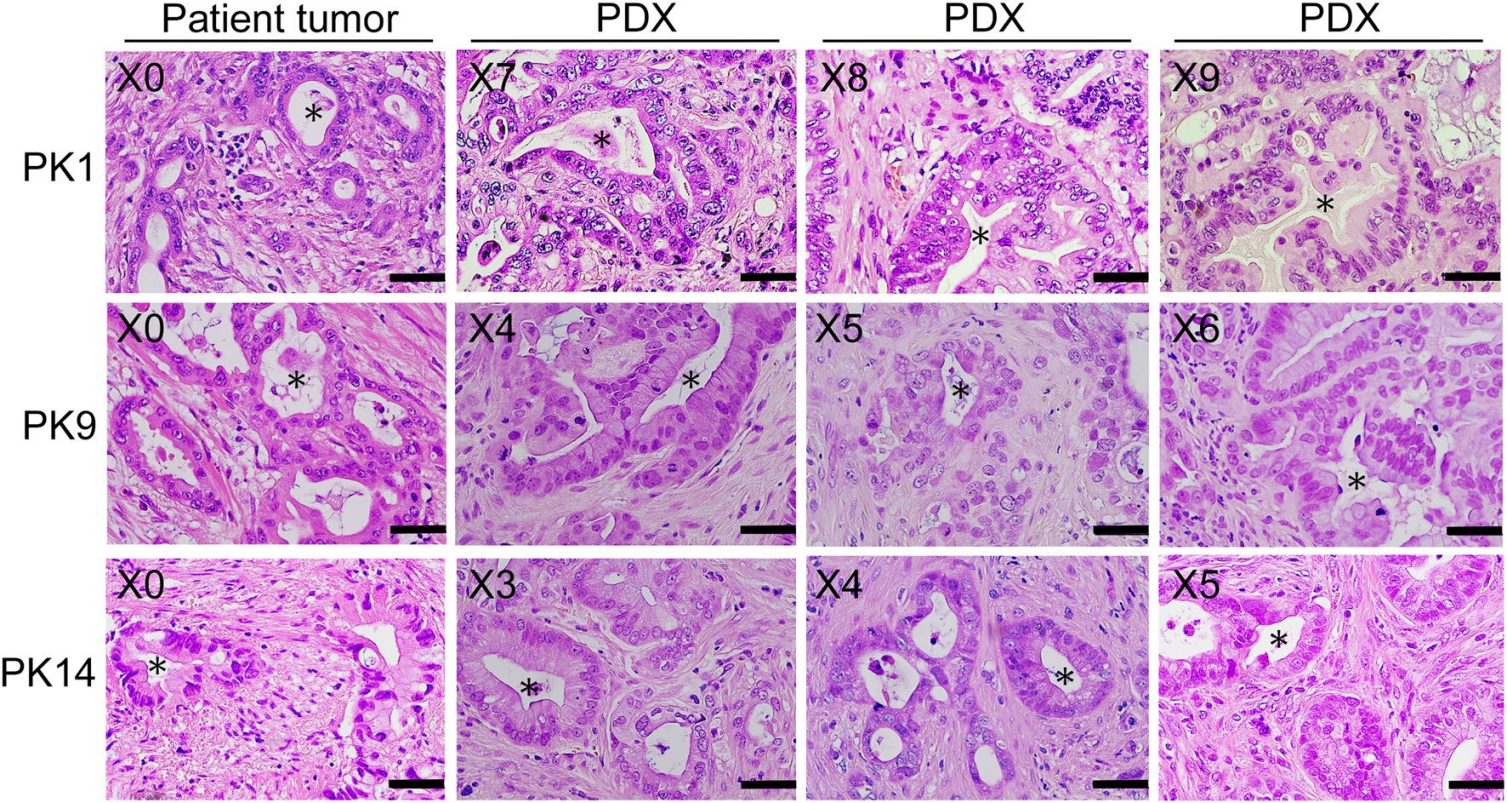
For three types of patient-derived tumors, representative examples of histopathological features using hematoxylin and eosin (HE) staining of original patient tumors and three consecutive generations of xenograft tumors corresponding to original patient tumors. Images are captured at a magnification of400X. *Scale bar* 50 μm. Original patient tumors are depicted in the first column from the left. The three consecutive generations of xenograft tumors, corresponding to original patient tumors, are depicted in the second to forth columns from the left. X with number in the upper left corner of each panel indicates the number of tumor generations. Ductal adenocarcinoma is indicated by asterisks. Note; PDX, patient-derived xenograft tumor.

The xenograft tumors histologically resembled the original PDAC obtained from patients on examination with HE staining. HE staining revealed that xenograft tumors in rat liver maintained the histopathological features of adenocarcinoma seen in the original patient tumors (Fig. 5). Serially passaged xenograft tumors also retained the same morphology as their original patient tumors in patients (Fig. 5). Necrotic or apoptotic areas were not seen in HE-stained slides in any of the three arms. Further immunohistochemical analysis was therefore performed as below.

### Immunohistochemistry

**Figure 6:**
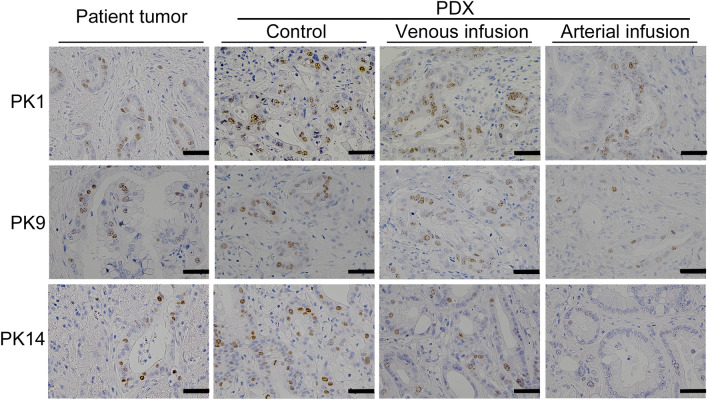
For three types of patient-derived tumors, representative examples of immunohistochemistry with Ki67 of original patient tumors and the corresponding xenograft tumors from the arterial infusion, the venous infusion, and the control groups. Images are captured at a magnification of 400X. *Scale bar* 50 μm. Original patient tumors are depicted in the first column from the left. The corresponding xenograft tumors are depicted in the second to forth columns from the left. The expression of Ki67 in the xenograft tumors of the control groups (second column from the left) is similar to that in original patient tumors (first column from the left), and is higher than that in the xenograft tumors of the arterial infusion groups (forth column from the left). Note; PDX, patient-derived xenograft tumor.

**Figure 7:**
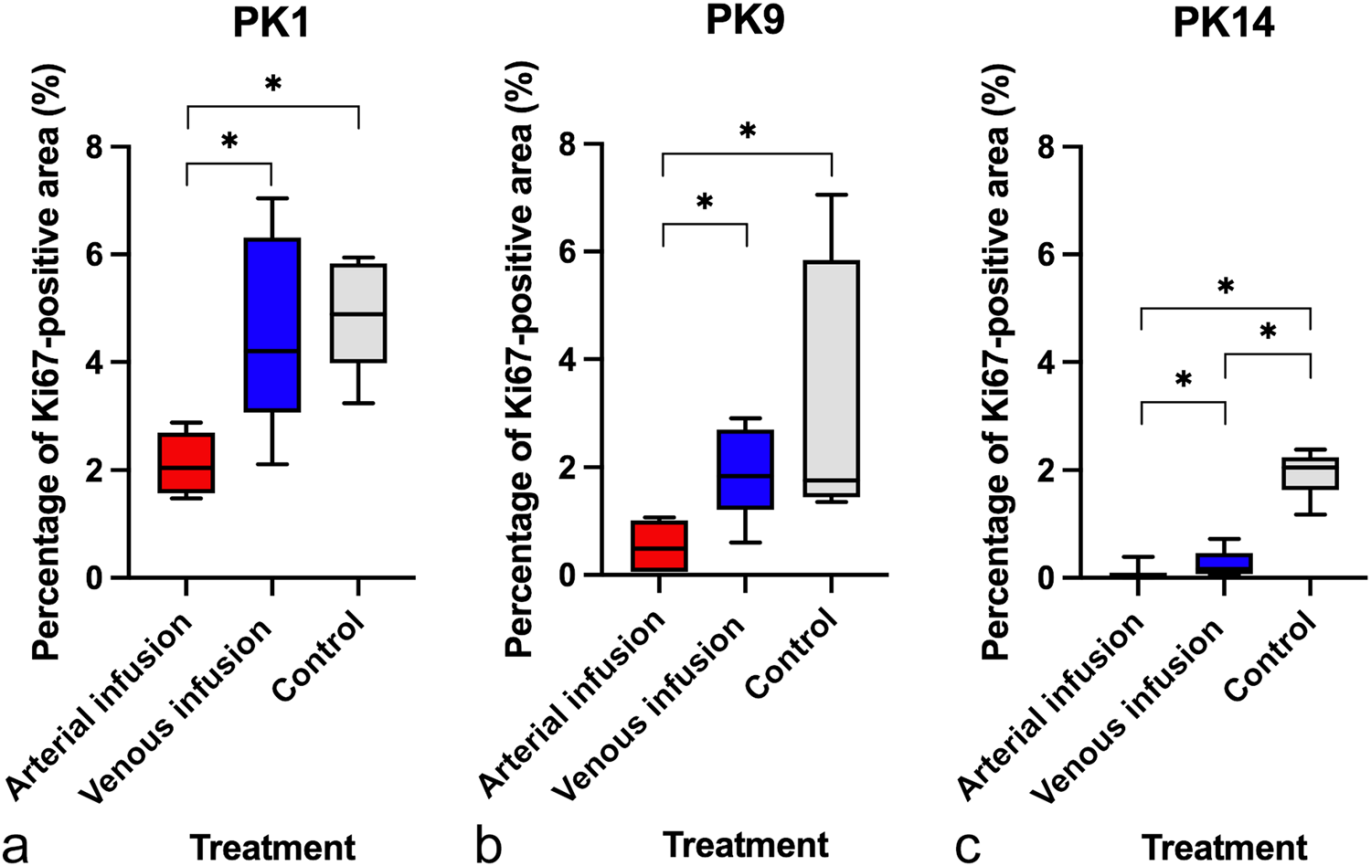
Mean percentage of Ki67-positive area at 4-weeks after treatment for three types of patient-derived tumors. (**a**) PK1. (**b**) PK9. (**c**) PK14. *p-value < 0.05.

The xenograft tumors in the control group retained a similar degree of Ki67 expression as that observed in the original patient tumors in all three cases (Fig. 6). The percentage of Ki67-positive area was significantly less in the arterial infusion group than in the venous infusion and control groups in all three cases (vs. venous infusion in PK1, *p* = 0.0041; vs. control in PK1, *p* < 0.001; vs. venous infusion in PK9, *p* = 0.004; vs. control in PK9, *p* < 0.001; vs. venous infusion in PK14, *p* = 0.038; vs. control in PK14, *p* < 0.001;) (Figs. 6 and 7). Collectively, these results indicate that high expression of Ki67 in tumors conferred enhanced tumor growth in PDX rats.

## Discussion

A rat-based platform for anticancer drug treatment has been successfully established. The liver-implanted PDAC PDX model was developed using severely immunodeficient rats with a success rate of 100%. Transcatheter hepatic arterial infusion chemotherapy and systemic venous infusion chemotherapy were administered to this PDX model. To assess the efficacy of cisplatin as an anticancer drug, comparisons were conducted between hepatic arterial infusion chemotherapy and systemic venous infusion chemotherapy. The venous infusion groups did not show inhibition of tumor growth, whereas the three arterial infusion groups showed antitumor effects of cisplatin, with one group showing tumor shrinkage. In other words, antitumor effects differed among the three patient-derived tumors. Clarification of differences in efficacy using the PDX model is likely to facilitate the creation of tailor-made therapies, although the present study used only one kind of anticancer drug. Taken together, these findings established a platform for preclinical trials using rat models.

This study was conducted on a liver-implanted PDAC PDX rat model with treatment methods, evaluation methods, and treatment schedules similar to those applied in human clinical practice. Mice are predominantly used as animal models in various experiments^31,32^, but show limitations with intravenous, oral, or abdominal delivery routes as drug administration methods^33,34^. Utilizing rats provides more options for drug administration and surgical treatment^35,36^. Nishiofuku et al. developed a technique for catheter insertion into a proper hepatic artery in rats^18^. In this study, we adopted their method to perform proper hepatic arterial infusion chemotherapy in rats. This study represents the first description of transcatheter hepatic arterial infusion chemotherapy conducted using a liver-implanted PDX rat model. Creation of liver metastases by injecting pancreatic cancer cells into the portal venous system would be the intuitive approach, but such a method results in the generation of numerous microscopic metastatic nodules^37^. This creates a situation inappropriate for the evaluation of anticancer drugs. Kageyama et al. previously developed a method of surgical liver implantation to embed a tumor chunk within the liver, forming a single intrahepatic nodule, as so-called “liver pocket method”^22^. To achieve uniformity of pre-treatment tumor size, that method of direct tumor implantation was adopted in the present study. Tumor volume was evaluated using CT in this study, mirroring the evaluation process in human clinical practice for anticancer drugs. With human anticancer therapy, CT is popularly used for evaluation as most tumors are deep-seated and not externally visible^38^. Nota et al. developed a minimally invasive method for evaluating rat liver tumors on contrast-enhanced CT using Exitron nano 12000 contrast agent in a rat liver tumor model^25^. They validated the concordance between CT images and tumor size in pathological specimens. Using the contrast agent, this study allowed the evaluation of tumor volume in the livers of living rats without sacrificing them by sequential CT scans before treatment and one month after treatment. The use of CT allowed long-term follow-up for a total of 8 weeks. The PDX model serves as a preclinical study platform for assessing antitumor efficacy in humans.

Hepatic arterial infusion with cisplatin demonstrated antitumor effects in a liver-implanted PDAC PDX rat model during this experiment. Cisplatin is not typically used as a standard drug for PDAC treatment. Typical treatment regimens for PDAC involve combination chemotherapy, such as FOLFIRINOX or gemcitabine plus nab-paclitaxel^5^. Previous clinical trials have investigated whether the addition of cisplatin to gemcitabine monotherapy improves survival^19,20^. Although no significant difference was identified, a survival benefit was observed with the combination of gemcitabine and cisplatin compared to gemcitabine monotherapy. When evaluating drugs in combination therapy, determining the effectiveness of cisplatin in providing a survival benefit is difficult if gemcitabine already offers sufficient benefit. In this context, the PDX model allowed assessment of the efficacy of single agents as anticancer drugs in this study. With regard to the route of drug administration, intraarterial administration of cisplatin resulted in increased exposure of the tumor to the administered drug in the infused area, without substantially decreasing the exposure of systemic tumor^39,40^. The rationale for hepatic arterial infusion chemotherapy is that cisplatin uptake by liver tumors is approximately 5–tenfold greater than that of venous infusion^6^. Hepatic arterial infusion of cisplatin for hepatocellular carcinoma is more effective than systemic infusion^41,42^. However, no reports have clarified the efficacy of hepatic arterial infusion chemotherapy with cisplatin as a single agent for PDAC liver metastasis. This study demonstrated the efficacy of intraarterial therapy with cisplatin as a single agent in a PDX model compared to systemic venous infusion. Hepatic arterial infusion with cisplatin is expected to be one modality that can provide significant antitumor effects against PDAC liver metastases.

With all three patient-derived tumors used in this study, both the original patient tumors and their corresponding xenograft tumors in the control group exhibited identical histological features in terms of HE staining and Ki67 immunohistochemistry. Surgical orthotopic implantation of PDACs into the liver thus offers a reliable method for creating PDX rat models that retain the characteristics of the original patient tumors. With PDX research, limiting the passage number in models is advisable to preserve the tumor characteristics of the original tumor^43^. Repeated serial passages can lead to genomic rearrangements intrinsic to tumor adaptation^44,45^. Generally, multiple passages in PDX models or changing the implantation site are not recommended to maintain the genetic and proteomic consistency of the original patient tumor^43,46^. In this study, we utilized PDX tumors that had undergone fewer than 10 passages. The expression rate of Ki67 was found to be closely associated with antitumor effects in this study. Ki67 is generally linked to cell proliferation and tumor progression^47^, and has been identified as an independent prognostic factor in PDAC^48^. Kageyama et al. reported a significant correlation between the Ki67 index and serial passaging, as well as successful engraftment^22^. Conversely, Pergolini et al. reported that successful engraftment of PDAC was associated with adverse clinicopathological features and poor survival^49^. In this study, a low expression rate of Ki67 was observed in the arterial infusion group, indicating high antitumor activity after treatment in PDAC.

Two key limitations to this study should be considered when interpreting the results. First, only one drug was utilized in the experimental setup. Standard chemotherapies such as FOLFIRINOX or gemcitabine plus nab-paclitaxel should be evaluated in the PDX model. However, the present study avoided the use of multiple drugs because of the difficulty in determining which specific drugs provided efficacy. Since the experimental system in this study represents an emerging method in the initial stage of evaluation, this study adopted cisplatin, as an established product for arterial infusion. To enable personalized chemotherapy in the near future, simultaneous examination of multiple drugs is essential to identify optimal anticancer drugs for individual patient treatments. Various kinds of anticancer drugs should be utilized in the near future. Second, this study included only three distinct PDACs from patients. Increasing the number of PDAC lines and utilizing a larger cohort of immunodeficient rats continue to present significant challenges.In, conclusion, a liver-implanted PDAC PDX rat model was established as a rat-based preclinical platform in a severely immunodeficient rat. This rat model enables assessment of the efficacy of anticancer drugs as preclinical studies representing humans. Chemotherapy comprising hepatic arterial cisplatin infusion offers a potential therapy to provide significant antitumor effects against PDAC liver metastases.

## Data Availability

The datasets generated or analyzed during the current study are available from the corresponding author upon reasonable request.

## Abbreviations

PDAC: Pancreatic ductal adenocarcinoma
FOLFIRINOX: Folinic acid, fluorouracil, irinotecan, and oxaliplatin
PDX: Patient-derived tumor xenograft
IL2Rg: Interleukin 2 receptor γ chain
X-SCID: X-linked severe combined immunodeficiency
PBS: Phosphate-buffered saline
HE: Hematoxylin and eosin
